# A systematic review and meta-analysis on correlation of weather with COVID-19

**DOI:** 10.1038/s41598-021-90300-9

**Published:** 2021-05-24

**Authors:** Poulami Majumder, Partha Pratim Ray

**Affiliations:** 1 Department of Biotechnology, Maulana Abul Kalam Azad University of Technology, Kolkata, India; 2 Department of Computer Applications, Sikkim University, Gangtok, India

**Keywords:** Climate sciences, Environmental sciences, Viral infection, Statistical methods, Software

## Abstract

This study presents a systematic review and meta-analysis over the findings of significance of correlations between weather parameters (temperature, humidity, rainfall, ultra violet radiation, wind speed) and COVID-19. The meta-analysis was performed by using ‘meta’ package in R studio. We found significant correlation between temperature (0.11 [95% CI 0.01–0.22], 0.22 [95% CI, 0.16–0.28] for fixed effect death rate and incidence, respectively), humidity (0.14 [95% CI 0.07–0.20] for fixed effect incidence) and wind speed (0.58 [95% CI 0.49–0.66] for fixed effect incidence) with the death rate and incidence of COVID-19 (*p* < 0.01). The study included 11 articles that carried extensive research work on more than 110 country-wise data set. Thus, we can show that weather can be considered as an important element regarding the correlation with COVID-19.

## Introduction

COVID-19 has impacted significantly over the human society in recent times^1–4^. More than 25 million population is already infected and over 0.8 million are already died of by the COVID-19^5^. Scientific organizations are currently involved in the development of possible vaccines to further stop the deadly spread of COVID-19^6–15^. Weather conditions always play important roles to the enhancement or eradication of health issues^16–19^. Thus, we can look for finding answer of the research question: whether weather has any correlation with COVID-19^20^**.**

A study^21^ was conducted to find the possibility of correlation between weather parameters with COVID-19. However, the comments didn’t conform to specific answer of weather impact on COVID-19. A study was conducted to test the impact of temperature on Australia and Egypt as a case study^22^. It suggested that there is a relation between temperature and COVID-19. A systematic review was performed where advocacy was made in favour of low evidence for impact of temperature and humidity on COVID-19^23^. No meta-analysis was done in this work. Harmooshi et al.^24^ investigated a generic review of 16 articles having some outcome-based impact over COVID-19. This work suggested that cool weather may affect transmissibility of COVID-19. In^25^, a prediction model was investigated for India in stating probable condition in 2020 due to COVID-19. Weather impact was found in Turkey over a 14-day long study^26,27^ suggested that incidence of COVId-19 could lower with high temperature and high wind speed. Thus, we can see that different articles stated their own point of view via various methods while resulting into confusion.

## Methods

In this paper, we present first ever meta-analysis of impacts of weather on the death and incidence on the COVID-19. Initially, we selected vital articles from digital repositories to find resourceful information. Thus, we performed a systematic review upon proper inclusion and exclusion criteria. Secondly, we used risk assessment of the included articles in this study. Thirdly, we performed evidence certainty tests of such articles to find suitability over the significant impact analysis of weather over COVID-19. We selected five weather parameter such as, temperature, humidity, rainfall, ultra violet and wind speed to find correlation with the death rate and incidence of the COVID-19. Fourthly, we performed forest and funnel plots to investigate the heterogeneity and publication bias, respectively.

### Search strategy

A comprehensive literature survey was conducted while considering articles from the following digital databases such as, PubMed, Sciencedirect, IEEE Xplore, Google Scholar, and Cochrane. We used a set of combination of key words to search the articles as shown in Table 1. One independent author (PPR) performed screening of abstract and titles of the literature against the aforementioned keyword and scope of the study. Other author (PM) did the review of final selection of the articles. Evaluation of full-texts were conducted against the inclusion and exclusion criteria.

**Table 1:** Keywords used for literature search.

Paper	Remarks on observations
PubMed	‘COVID-19’, ‘COVID-19’ AND ‘Weather’, ‘COVID-19’ AND ‘Weather’ AND ‘Impact’, ‘COVID-19’ AND ‘Weather’ AND ‘Correlation’, ‘COVID-19’ AND ‘Correlation’
Sciencedirect	‘SARS-COV-2’ AND ‘Weather’ AND ‘Correlation’, ‘SARS-COV-2’ AND ‘Correlation’, ‘COVID-19’ AND ‘Temperature’ AND ‘Correlation’, ‘COVID-19’ AND ‘Humidity’ AND ‘Correlation’
IEEE Xplore	‘COVID-19’ AND ‘Weather’ AND ‘Correlation’, ‘COVID-19’ AND ‘Correlation’, ‘SARS-COV-2’ AND ‘Weather’, ‘SARS-COV-2’ AND ‘Weather’ AND ‘Impact’
Google scholar	‘SARS-COV-2’ AND ‘Weather’, ‘SARS-COV-2’ AND ‘Weather’ AND ‘Impact’, ‘SARS-COV-2’ AND ‘Weather’ AND ‘Correlation’, ‘SARS-COV-2’ AND ‘Correlation’, ‘COVID-19’ AND ‘Temperature’ AND ‘Correlation’, ‘COVID-19’ AND ‘Humidity’ AND ‘Correlation’, ‘COVID-19’ AND ‘UV’ AND ‘Correlation’, ‘COVID-19’ AND ‘Rainfall’ AND ‘Correlation’, ‘COVID-19’ AND ‘Wind’ AND ‘Correlation’, ‘COVID-19’ AND ‘Weather’ AND ‘Correlation’ AND ‘Meta-analysis’, ‘COVID-19’ AND ‘Weather’ AND ‘Correlation’, AND ‘Review’
Cochrane	‘SARS-COV-2’ AND ‘Weather’, ‘SARS-COV-2’ AND ‘Weather’ AND ‘Impact’, ‘SARS-COV-2’ AND ‘Weather’ AND ‘Correlation’, ‘SARS-COV-2’ AND ‘Correlation’

### Study selection

**Figure 1:**
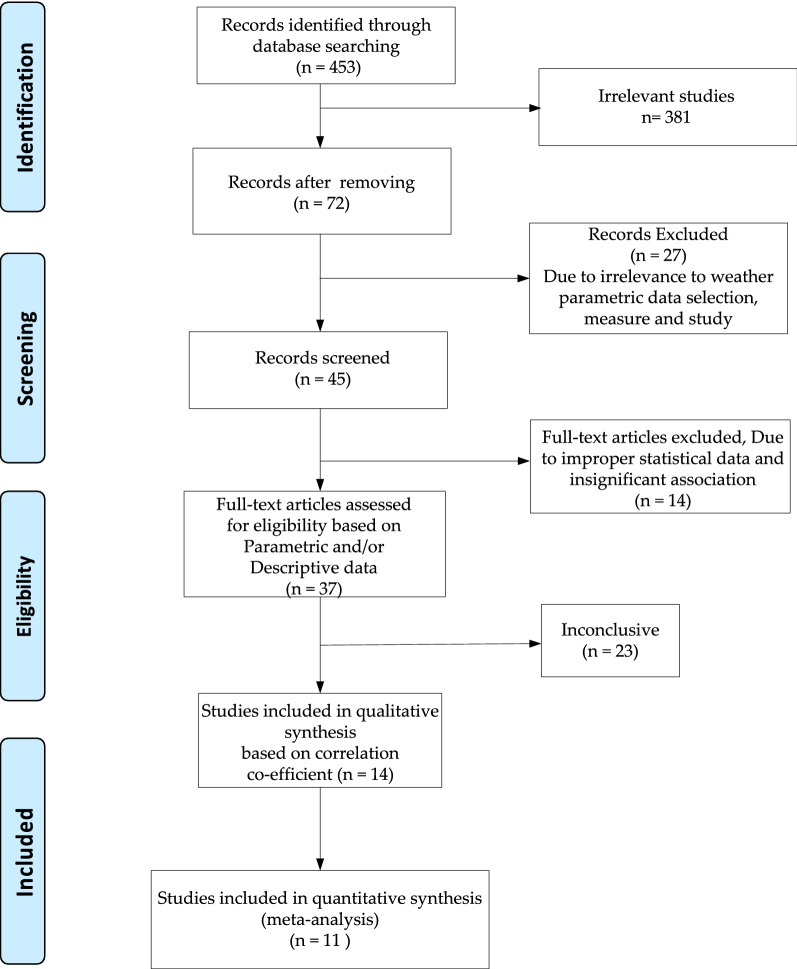
PRISMA flowchart for the study.

The work was done as per the Preferred Reporting Items for Systematic Reviews and Meta-Analyses (PRISMA) guidelines^28^. We conducted a qualitative analysis of the 11 included articles in this study based on publication year, zone or country of work, various variables used, key techniques used and remarks on the observations. Figure 1 presents the PRISMA of the meta-analysis. Inclusion of articles depends on the availability of correlation factors in the surveyed articles. We have included those studies that only discusses about the correlation between weather parameters to COVID-19. We also, seek for the relevance of performed studies in the article to prescribe some key suggestions. Further, we include those articles that are full-text published but not from the medRxiv repository for meta-analysis. We focused on the quantitative synthesis of statistical approaches used in the articles. We excluded all the articles which are published in non-indexed journals and don’t conform to the direct correlation perspective of COVID-19 with weather factors. Due to lack of minimal availability, we exclude the correlating parameters related to the pollution, air quality index (AQI), pollination, and sun light intensity as the weather parameters in this meta-analysis.

**Figure 2:**
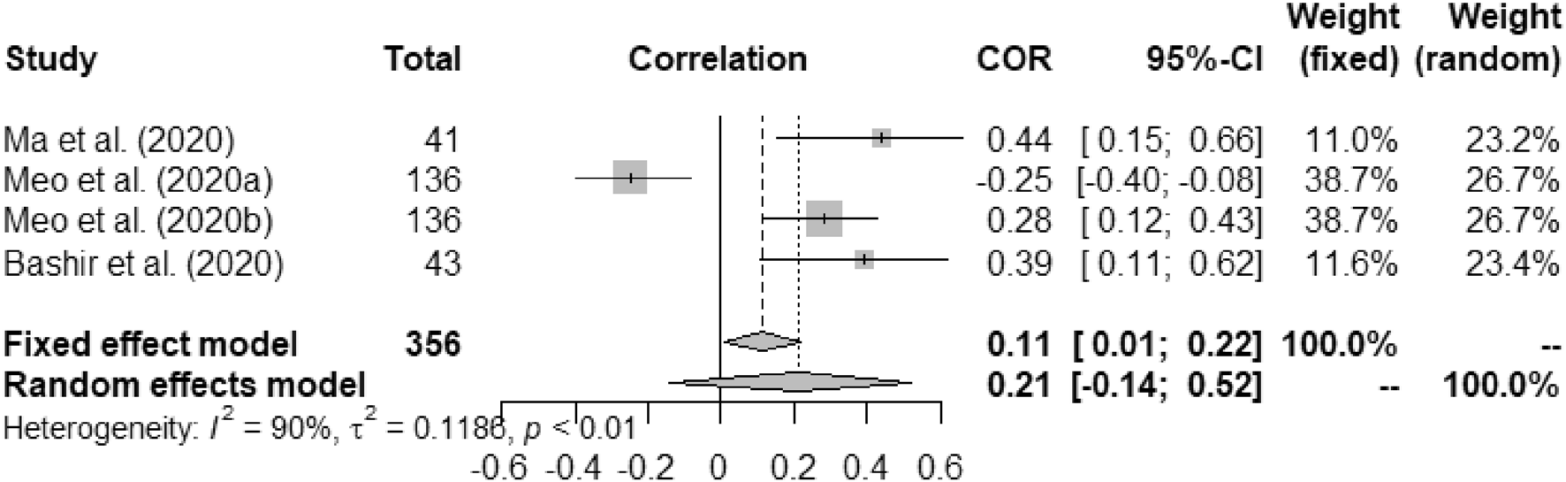
Forest plot of COVID-19 death rate with temperature.

### Assessment of risk of bias

We assessed the quality of the articles selected in this study by using the Joanna Briggs Institute (JBI) tool^29^. The checklist contained eight questions such as (a) were the criteria for inclusion in the sample clearly defined, (b) were the study subjects and the setting described in detail, (c) was the exposure measured in a valid and reliable way, were objective, (d) standard criteria used for measurement of the condition, (e) were confounding factors identified, (f) were strategies to deal with confounding factors stated, (g) were the outcomes measured in a valid and reliable way and (h) was appropriate statistical analysis used. Each of the question was examined against each of the 11 articles and answer was given in ‘Yes’ and ‘No’. Overall risk was finally specified at the bottom of Table 2 with two main answers such as, ‘Low’ and ‘Moderate’. Both the authors (PPR and PM) independently evaluated risk and quality of each study and confusion was mitigated by a consensus team meeting.

**Table 2:** Risk bias assessment of the literature included in this study.

Questions/Paper	Ma et al.	Wang et al.	Islam et al.	Qi et al.	Meo et al.	Rashed et al.	Tosepu et al.	Bashir et al.	Vinoj et al.	Sajadi et al.	Xu et al.
1. Were the criteria for inclusion in the sample clearly defined?	Yes	Yes	Yes	Yes	Yes	Yes	Yes	Yes	Yes	Yes	Yes
2. Were the study subjects and the setting described in detail?	Yes	Yes	No	Yes	Yes	Yes	Yes	Yes	Yes	Yes	Yes
3. Was the exposure measured in a valid and reliable way?	Yes	Yes	No	No	Yes	Yes	No	No	No	No	Yes
4. Were objective, standard criteria used for measurement of the condition?	Yes	Yes	No	No	Yes	Yes	No	Yes	No	No	Yes
5. Were confounding factors identified?	Yes	Yes	No	No	No	Yes	Yes	No	Yes	No	Yes
6. Were strategies to deal with confounding factors stated?	Yes	Yes	No	No	No	Yes	Yes	No	Yes	No	Yes
7. Were the outcomes measured in a valid and reliable way?	Yes	Yes	Yes	Yes	Yes	Yes	No	Yes	Yes	No	Yes
8. Was appropriate statistical analysis used?	Yes	Yes	Yes	Yes	Yes	Yes	Yes	Yes	Yes	Yes	Yes
Risk of Bias	Low	Low	Moderate	Moderate	Moderate	Low	Low	Low	Moderate	Moderate	Low

### Data extraction and outcome measure

Data was extracted for following variables such as, (a) temperature, (b) humidity, (c) rainfall, (d) ultra violet (UV) radiation and (e) wind speed. We considered two key COVID-19 parameters such as, (a) death rate and (b) incidence. Thus, five key weather elements were used to find association with two COVID-19 parameters for performing meta-analysis on possible weather impact on COVID-19. Solar radiation and UV radiation were assumed to be same by considering SI unit i.e. W-m^-2^. We considered relative humidity out of absolute and relative humidity while performing this meta-analysis. Major characteristics of the included studies rely in the recently performed correlation assessment between the weather parameters with the incidence or death rate of COVID-19. Further, we considered the evaluation criteria as mentioned in the articles to provide the meta-analysis.

### Certainty measure

The GRADE (Grading of Recommendations Assessment, Development, and Evaluation)^30^ approach was used to evaluate the quality of evidence for each outcome as shown in Table 3. We tested 7 outcomes on the correlations between (a) temperature and COVID-19 death rate, (b) humidity with COVID-19 death rate, (c) temperature with COVID-19 incidence, (d) humidity with COVID-19 incidence, (e) rainfall with COVID-19 incidence, (f) UV with COVID-19 incidence, and (g) wind speed with COVID-19 incidence. We found the impact of each of the outcomes. We also measured the evidence of certainty using ⊕ AND/OR◯ combination of four symbols in terms of ‘Moderate’, ‘High’, and ‘Very High’. The points in the GRADE analysis are considered as follows. Very High point is given to the correlation factor that shows the highest order significance among all the included works. Similarly, High point is given to those parametrization aspects where we notice strong evidence of measure. We give Moderate as the lowest measure to the correlating perspective having lowest of significance.

**Table 3:** GRADE evidence profile table.

Outcomes	Impact	Number of studies	Certainty of the evidence (GRADE)
Correlation between temperature with death rate of COVID-19	Out of three articles, two showed positive correlation of temperature with death rate of COVID-19. One article showed negative correlation with the death rate with COVID-19 i.e. in hottest countries.	(3 OBSERVATIONAL STUDIES)	⊕⊕◯◯ Moderate
Correlation between humidity with death rate of COVID-19	Out of three articles, all papers showed negative correlation of humidity with death rate of COVID-19.	(3 OBSERVATIONAL STUDIES)	⊕⊕⊕⊕ Very high
Correlation between temperature with incidence of COVID-19	Out of ten articles, seven showed positive correlation of temperature with incidence of COVID-19. Three articles that covered most of the hot countries in their study revealed negative correlation with incidence of COVID-19 with average yearly temperature.	(10 OBSERVATIONAL STUDIES)	⊕⊕⊕◯ High
Correlation between humidity with incidence of COVID-19	Out of ten articles, four showed negative correlation of humidity with incidence of COVID-19. Three articles revealed positive correlation with incidence of COVID-19. Studies of Meo et al. in both hot and cool countries showed negative correlation.	(10 OBSERVATIONAL STUDIES)	⊕⊕◯◯ Moderate
Correlation between rainfall with incidence of COVID-19	Two out of three studies showed positive correlation between rainfall and incidence of COVID-19. One study showed negative correlation.	(3 OBSERVATIONAL STUDIES)	⊕⊕◯◯ Moderate
Correlation between UV with incidence of COVID-19	One out of two studies showed high negative correlation between UV radiation and incidence of COVID-19. Overall correlation is negative.	(2 OBSERVATIONAL STUDIES)	⊕⊕⊕◯ High
Correlation between windspeed with incidence of COVID-19	All of three articles showed positive correlation between windspeed and incidence of COVID-19.	(3 OBSERVATIONAL STUDIES)	⊕⊕⊕⊕ Very high

### Statistical analysis

Accessed data from 11 articles were initially recorded into the excel datasheet which was later segregated into 7 different comma separated value CSV) files for feeding into the RStudio version 3.4.3 with package meta. We used metacor(cor = r, n = N, data = d, studlab = Author, sm = "ZCOR") method call to perform the fixed-effect and random effect model study. We used Fisher’s z transformed correlations to find meta-analysis. Here, r, N and d represent the CSV columns named as r, N and the CSV itself, respectively. Where, r and N (sample size) of the specific CSV stored the correlation values in ( +) and/or (-) terms and days of experiment by individual article, respectively. 95% confidence interval (CI) was measured for each of the articles. Wang et al. (2020a), Wang et al. (2020b), Meo et al. (2020a), and Meo et al. (2020b) were sub-set wise used of the Wang et al. (2020) and Meo et al. (2020) articles, respectively. Fixed and random weight of each of the article was computed. We found heterogeneity (I^2^) and τ^2^ as the level of heterogeneity and measure of dispersion of true effect sizes under the given assumptions that the true effect sizes were bell-shaped and normally distributed, respectively. We used the forest() method to derive the forest plots for seven different scenarios of correlation meta-analysis with help of the Fisher's z transformed correlations.

## Results

### Study selection and characteristics

The article reporting and record keeping task was finalized on August 6, 2020. All the included papers belong to the initial to recent COVID-19 impacts i.e. December 1, 2019–June 5, 2020. Based on initial record screening, we found 453 articles. We remove 381 irrelevant articles and later moved with 72 records. Due to irrelevance to weather parametric data selection, measurement and study approaches, we excluded 27 articles. Out of 45 articles, upon full-text screening we found improper statistical data and insignificant association between weather and COVID-19, we rejected 14 articles. Rest of the 37 articles were focused on wither parametric or description statistical association study between the weather and COVID-19. However, 23 were found to be nonconclusive toward correlation between weather and COVID-19 which were later on rejected. Out of 14 articles, only 11 were finally included in this meta-analysis. All the studies discussed about some sort of correlation factor with one or more weather parameters comprising of temperature, humidity, rainfall, UV and wind speed with the COVID-19 death rate or incidence level in various parts of globe. The articles conducted studies in different zones of countries belonging to Wuhan, China, mainland China, India, USA, Japan, Jakarta, Indonesia, Australia, Canada, Iran and more than 110 countries. The article mainly used the Pearson’s correlation coefficient, cohort study, Spearman’s rank correlation logarithmic estimation, generalized additive model (GAM) and Fama–Macbeth regression statistical techniques. Out of 11 only 1 article remarked about the basic reproduction number i.e. R_0_ in conjunction to the weather parameters for possible impact on the COVID-19 incidence.

### Survey of articles

Table 4 presents the comparison between the articles included in this study. Wang et al. (2020a) and Wang et al. (2020b) represent a single article but two different works related to China and USA. Similarly, Meo et al. (2020) performed studies on 10 hottest and 10 coolest countries, thus two versions of citations were used into the further works such as Meo et al. (2020a) and Meo et al. (2020b) representing hot and cool countries, respectively.

**Table 4:** Comparison of literature included in this study.

Paper	Year	Zone/country	Variables	Time period	Technique used	Remarks on observations
Ma et al.^31^	2020	Wuhan, China	Temperature, Diurnal Temperature Range (DTR), Relative Humidity, Absolute Humidity, Air Pollutants	20 January, 2020–29 February, 2020	Generalized Additive Model	Correlation between COVID-19 death rate and weather parameters, positive correlation with DTR and negative with humidity
Wang et al.^32^	2020	China, USA	Temperature, Relative Humidity, Population Density, GDP per capita, Fraction of population aged $\ge$ 65 years	19 January, 2020–10 February, 2020, 15 March, 2020–25 April, 2020	Effect on basic reproductive number (R_0_), Fama-Macbeth Regression	Estimated that R_0_ declines about 0.89 in total, temperature and humidity play important role to reduce R_0_ of COVID-19
Islam et al.^33^	2020	310 Regions of 116 County	Temperature, Relative Humidity, Wind Speed	8 January, 2020–12 March, 2020	Estimation of adjusted incidence rate ratio (IRR)	Temperature, relative humidity, and wind speed has low incidence of COVID-19
Qi et al. ^34^	2020	China	Temperature, Relative Humidity	1 December, 2019–11 February, 2020	Generalized Additive Model, Exponential Moving Average	Significant negative association between the temperature and humidity with the COVID-19
Meo et al.^35^	2020	10 Hottest Countries, 10 Coolest Countries	Temperature, Relative Humidity	29 December, 2019–12 May, 2020	Descriptive Statistics	Significant decrease in in death rate and daily cases in hot countries than cool countries
Rashed et al.^36^	2020	16 Prefecture, Japan	Temperature, Relative Humidity	16 April, 2019–25 May, 2020	Spearman’s Rank Correlation	Impact of multivariate parameters on COVID-19
Tosepu et al.^37^	2020	Jakarta, Indonesia	Temperature, Relative Humidity, Rainfall	1 January, 2020–29 March, 2020	Spearman’s Rank Correlation	Temperature is significantly correlated with COVID-19 daily cases
Bashir et al.^38^	2020	New York, USA	Temperature, Relative Humidity, Rainfall, Wind Speed, Air Quality	1 March, 2020–12 April, 2020	Kendall’s Rank Correlation, Spearman’s Rank Correlation	Temperature, humidity and air quality significantly associated with COVID-19 death rate and daily cases
Vinoj et al.^39^	2020	Delhi, India	Temperature, Relative Humidity, Specific Humidity, UV Radiation	20 April, 2020–5 June, 2020	Pearson’s Correlation	Positive correlation with temperature and negative correlation with humidity and UV radiation in COVID-19
Sajadi et al.^40^	2020	50 Cities One Each from 50 Countries	Temperature, Relative Humidity	1 January, 2020–10 March, 2020	Cohort Study	Correlation with temperature and humidity was observed in COVID-19
Xu et al.^41^	2020	3739 Locations from Australia, China, Canada, Iran, USA	Temperature, Relative Humidity, Rainfall, Wind Speed, UV Radiation, O_3_, SO_2_, DTR, Air Pressure	12 December, 2019–22 April, 2020	Logarithmic Estimation	Relationship with temperature, humidity, rainfall, windspeed, UV radiation found with incidence of COVID-19

### Overall outcomes

Table 5 presents overall outcome from this study. Correlation between the temperature and COVID-19 death rate was measured as (a) fixed effect model: 0.11 (95% CI, 0.01–0.22) and (b) random effect model: 0.21 (95% CI − 0.14–0.52) with *p* < 0.01. Similarly, humidity and COVID-19 correlation were measured as − 0.13 (95% CI, − 0.23- 0.03) and − 0.13 (95% CI, − 0.23–0.03) for fixed and random effect model, respectively against p-value at 0.53.

**Table 5:** Overall outcome.

Outcome	Sample size	COVID-19 parameters	Pooled Correlation (95% CI)		I^2^ (%)	τ^2^	*p* Value
			Fixed effect model	Random effect model			
Temperature	356	Death rate	0.11 (0.01−0.22	0.21 (− 0.14–0.52)	90	0.1186	<0.01
	897	Incidence	0.22 (0.16–0.28)	0.23 (0.01–0.42)	90	0.1312	<0.01
Humidity	356	Death rate	− 0.13 (− 0.23- 0.03)	− 0.13 (− 0.23–0.03)	0	0	0.53
	897	Incidence	0.14 (0.07–0.20)	0.16 (− 0.20–0.48)	96	0.3936	<0.01
Rainfall	265	Incidence	0.04 (− 0.09–0.16)	0.03 (− 0.10–0.17)	16	0.0025	0.3
UV	187	Incidence	− 0.09 (− 0.23–0.06)	− 0.14 (− 0.43–0.18)	74	0.0394	0.05
Wind Speed	241	Incidence	0.58 (0.49–0.66)	0.62 (− 0.17–0.92)	98	0.6116	<0.01

In case of weather and COVID-19 incidence correlation aspect, we found that temperature had 0.22 (95% CI, 0.16–0.28) and 0.23 (95% CI, 0.01–0.42) for fixed and random study, respectively. We found that humidity had positive correlation with the COVID-19 incidence at *p* < 0.01. Rainfall had minimal positive correlation with COVID-19 incidence having 0.04 (95% CI, − 0.09–0.16)0.03 (95% CI, − 0.10–0.17) for fixed and random, respectively. Correlation between UV and COVID-19 incidence was measured as − 0.09 (95% CI, − 0.23–0.06) for fixed and − 0.14 (95% CI, − 0.43–0.18) for random model. Wind speed was found to have positive correlation with the incidence of COVID-19 such as, 0.58 (95% CI, 0.49–0.66) and 0.62 (95% CI, − 0.17–0.92).

Heterogeneity (I^2^) was mostly observed with the temperature, humidity (COVID-19 incidence) and wind speed variables i.e. 90%, 96% and 98%, respectively. Complete homogeneity i.e. (I^2^ = 0) was found in the humidity with the death rate of COVID-19 with zero τ^2^. I^2^ of rainfall was found as 16% against the COVID-19 incidence.

**Figure 3:**
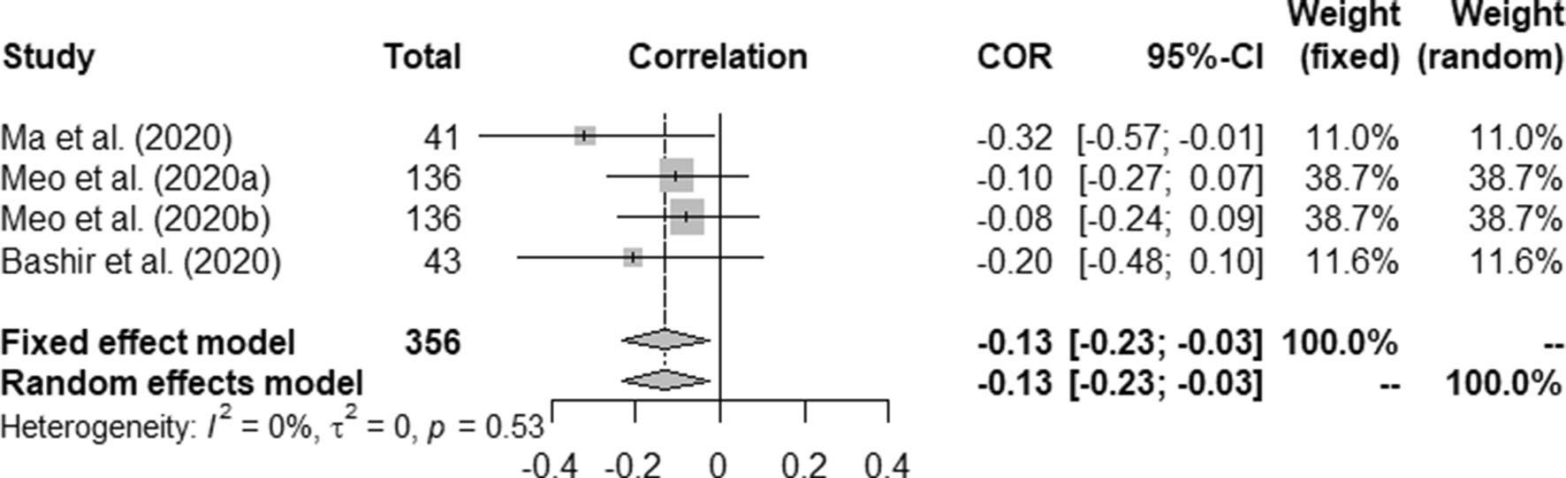
Forest plot of COVID-19 death rate with humidity.

**Figure 4:**
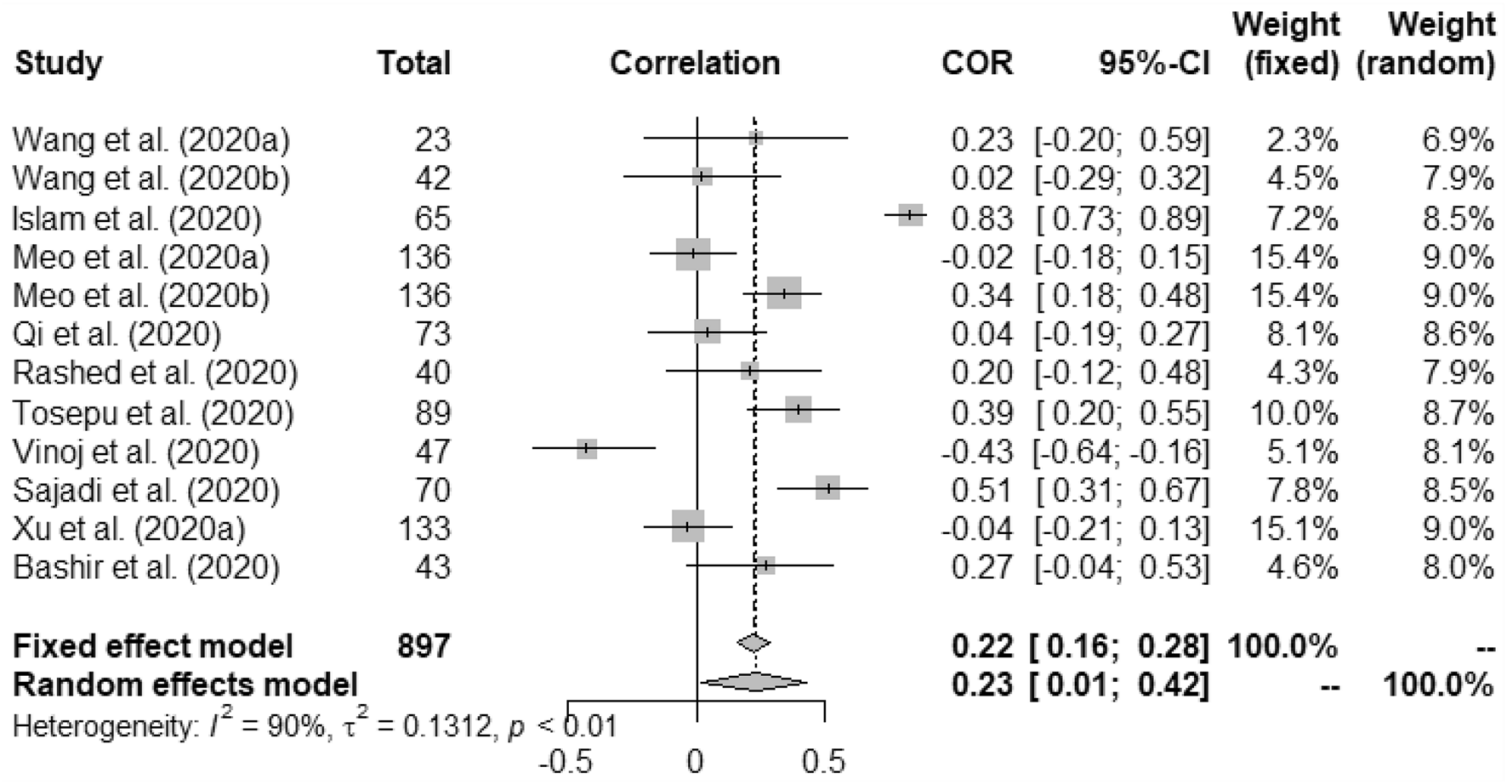
Forest plot of COVID-19 incidence with temperature.

**Figure 5:**
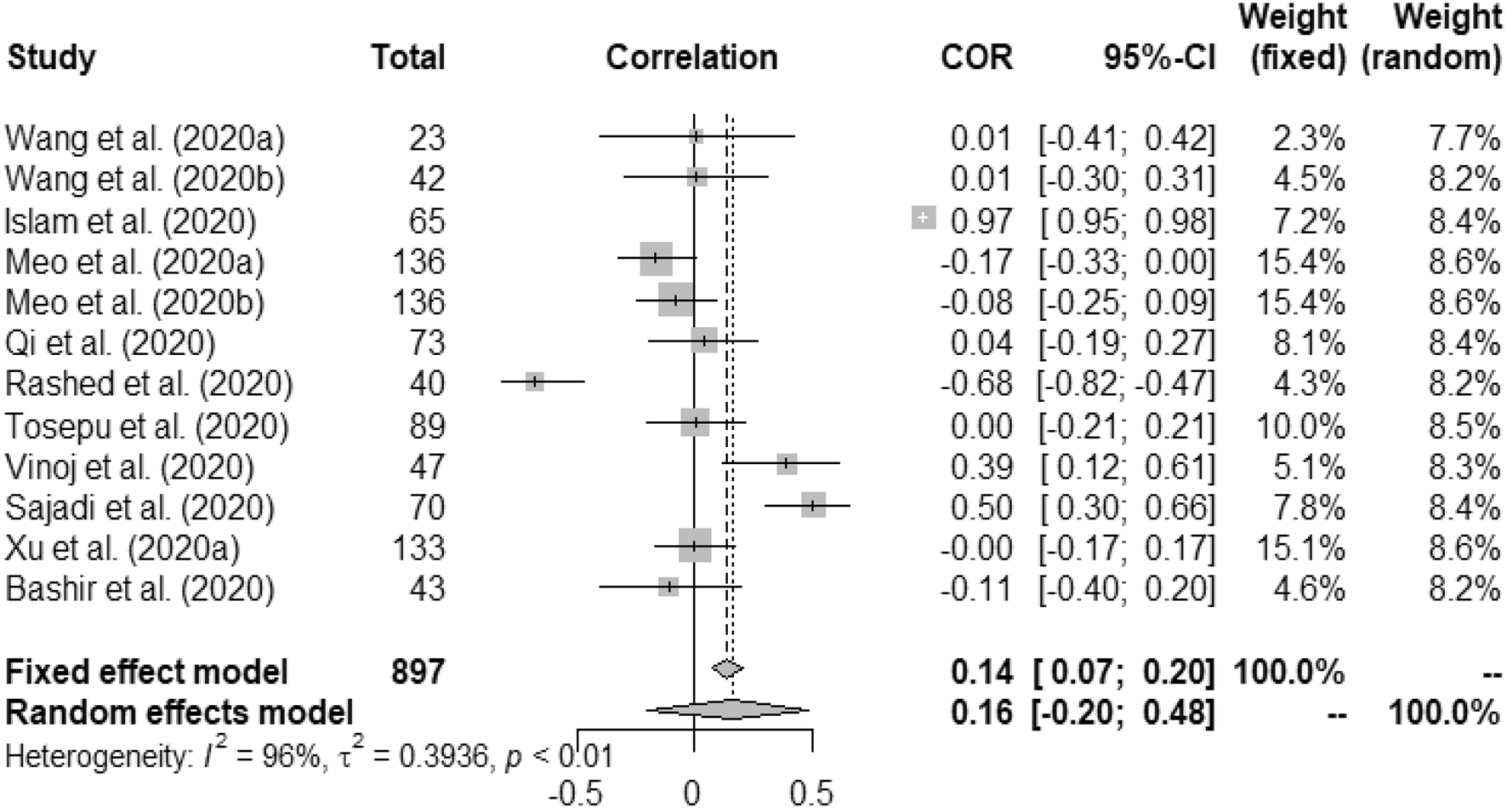
Forest plot of COVID-19 incidence with humidity.

**Figure 6:**
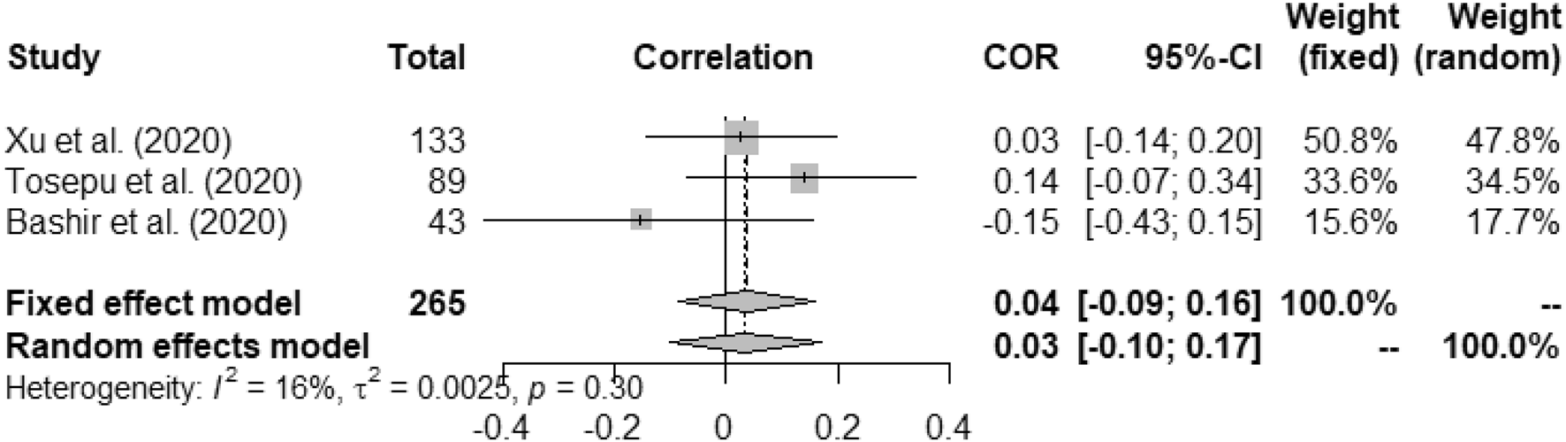
Forest plot of COVID-19 incidence with rainfall.

**Figure 7:**
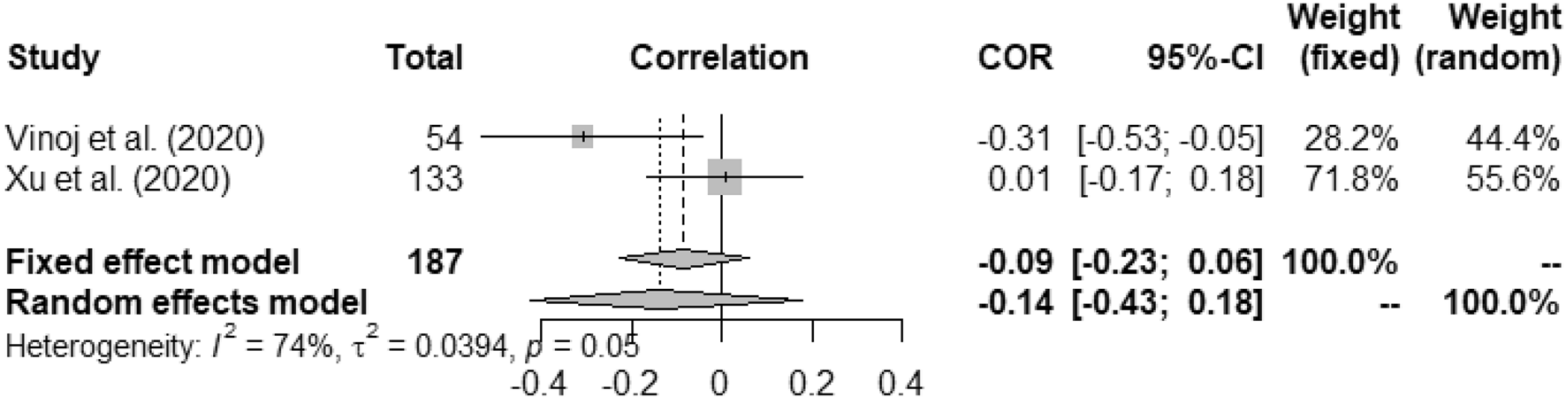
Forest plot of COVID-19 incidence with UV.

**Figure 8:**
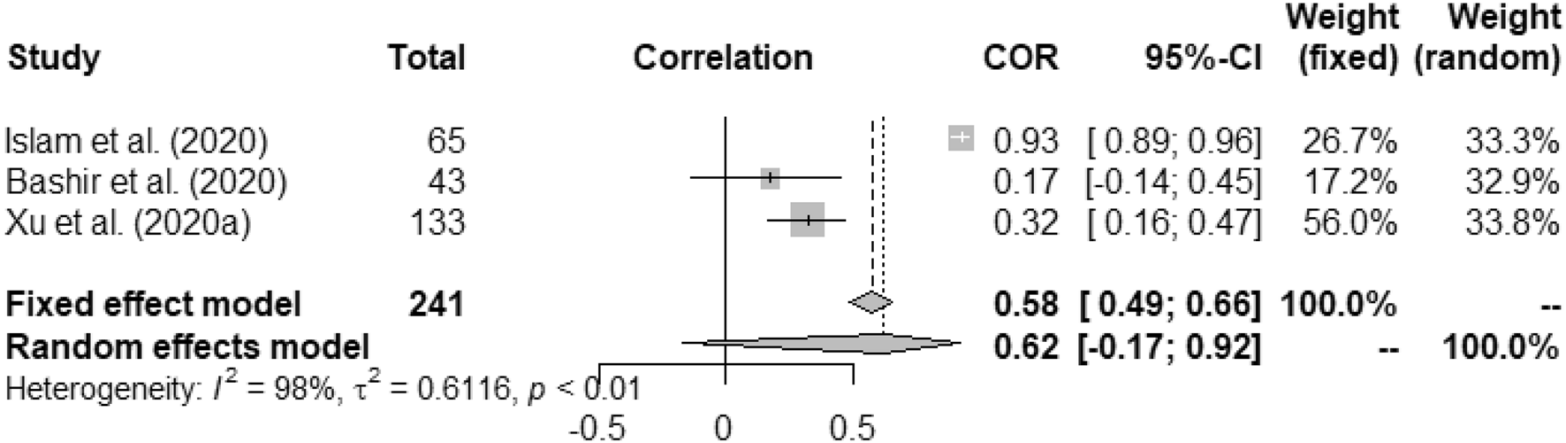
Forest plot of COVID-19 incidence with wind speed.

Figures 2, 3, 4, 5, 6, 7, and 8 present the forest plots of seven different correlation aspects of weather parameters with COVID-19 death rate and incidence.

## Discussion

To best of our knowledge, herein presented systematic review and meta-analysis is the first ever work to find answer of correlation between weather on COVID-19. Our meta-analysis is the first to analyse the effect of weather on the death rate and incidence of COVID-19. Based on our meta-analysis we found correlation between weather on the COVID-19. Temperature and humidity are most crucial weather factors that are string enough to impact over the death rate and incidence of COVID-19^42,43^. All the articles included into this study adhere to the weather centric approaches to the COVID-19. All the articles performed their research during December, 2019 to June, 2020. Thus, a long-time duration was covered in our meta-analysis to come at genuine and effective conclusion about possibility of weather impact on COVID-19. Correlation parameters were used in this study to disseminate direct relationship between the weather and COVID-19.

Our meta-analysis included more than 110 country data regarding weather impact on the coronavirus spread and deaths. As the articles carries extensive research during initial phase and mid phase of COVID-19 in most of the countries, this meta-analysis is far more effective to provide more specific answer to correlation-related questions which were frequently asked in near past. With involvement of the JBI tools and GRADE evidence profile, presented meta-analysis serves as an indispensable literature in the current context of COVID-19 incidence.

In this meta-analysis, we assumed the correlation values to be most effective than other alternatives due to its straight forward nature of relationship measurement approach. We depended our study over the fixed and random effect models asides the heterogeneity and dispersion of true size effects. Significant forest plots were obtained for the (a) temperature versus death rate, (b) temperature versus incidence, (c) humidity versus incidence, and (d) wind speed versus incidence of COVID-19 i.e. air borne. Though, impact of UV radiation over the incidence of COVID-19 was computed but negative correlation was observed. It means that with more UV radiation lesser incidence of COVID-19 can be found. Similarly, rainfall has a positive correlation with COVID-19 incidence.

We didn’t know the exact reason why such behaviour i.e. non-significance was observed. We can hypothesize that higher rainfall increases relative humidity in air thus a greater number of cases can be seen due to COVID-19. One surprising result was found in our meta-analysis i.e. negative correlation between humidity with death rate, though its relationship to the incidence was earlier discussed to be positively correlated. We not clear about the reason behind such nature of humidity.

Our work has some limitations including availability of plentiful research on weather correlation with COVID-19. This study restricted us to conduct meta-analysis on available articles where some of them were taken from various preprint servers. Thus, risk of rejection of those articles were not accurately considered, even though we used JBI and GRADE methods. We can also say that hot countries with high average temperature and relative humidity are more prone get affected by new incidences of COVID-19 in coming days. It can be estimated that during coming winter may provide some relief to the people of world. However, more research should be conducted to better support our meta-analysis conclusions.

## Conclusion

We found some strong correlations between weather over the incidence of COVID-19. The met-a analysis can be useful for the policy makers of the government and health incorporations to take prior decisions before the possible surge of COVID-19 cases depending on the weather forecasting mechanism. We urge the medical professionals and weather analysts to further investigate the findings of this article as the a-priori information to mitigate the COVID-19 pandemic.
